# O07 Managing relapsing and refractory lupus nephritis in juvenile systemic lupus erythematosus: a case report

**DOI:** 10.1093/rap/rkab067.006

**Published:** 2021-10-19

**Authors:** Farhan Javed, Harris Fayyaz, Eslam Al-Abadi, Warren Pickering, Maria Leandro, Rachel Jeffery

**Affiliations:** 1 Northampton General Hospital, Northampton, United Kingdom; 2 Birmingham Children Hospital, Birmingham, United Kingdom; 3 University College London Hospital, London, United Kingdom

## Abstract

**Case report - Introduction:**

Juvenile-onset systemic lupus erythematosus (jSLE) is a rare systemic autoimmune-disease affecting children, with an incidence between 0.5 and 6 per 100,000.

A 20-year-old female with known jSLE was diagnosed six years ago, age 14, following multiple attendances to A&E with non-specific symptoms. She was initially discharged with simple analgesia but later admitted to the paediatric ward with severe central chest pain. Investigations confirmed pleuro-pericardial effusion and positive lupus antibodies.

She subsequently had recurrent flares with predominantly renal involvement. She remained symptomatic despite multiple combined immunosuppressive agents including biological therapies. Unfortunately, her kidney-functions deteriorated, requiring close monitoring in the Advanced Kidney-Care Clinic.

**Case report - Case description:**

A 14-year-old female who was otherwise fit and healthy with no family history of autoimmune conditions presented to the Emergency department with constant throbbing limb-pain and recurrent episodes of chest pains.

Immunological investigations revealed positive anti-nuclear antibodies (ANA), anti-double-stranded(ds)-DNA antibodies, C1Q-antibody and lupus inhibitor on two repeat tests. Echocardiogram showed pleuro-pericardial effusion and due to the high risk of cardiac tamponade, she was transferred to a tertiary centre for further management. She had intravenous steroid with good resolution of symptoms, and subsequently received maintenance therapy of azathioprine and hydroxychloroquine. She stayed in remission for approximately one year before her clinical condition worsened. She started to develop active inflammatory arthritis and serositis, with increasing anti-dsDNA titres, reducing complement levels, worsening anaemia and a positive direct antiglobulin test. Oral corticosteroids were recommenced and azathioprine was switched to mycophenolate-mofetil.

She subsequently had recurrent flares; predominantly renal involvement and worsening proteinuria. This did not improve with repeat courses of antibiotics given for presumed urinary tract infections (UTIs), prompting renal biopsy which confirmed active renal lupus (combined Class III and V lupus nephritis). Rituximab and tacrolimus were initiated but rituximab was withheld after the first dose due to severe, widespread desquamating rash, mucosal ulceration and fever, biopsy-proven and drug-induced. Short-term remission after 1 dose of rituximab and then renal relapse with repeat renal biopsy showed class 5 lupus nephritis. Intravenous cyclophosphamide with Euro-lupus regimen was given.  5 months after discontinuation of cyclophosphamide her renal disease relapsed again with rising anti-dsDNA, ESR and proteinuria to > 6g/l, despite mycophenolate. Repeat and extended course of cyclophosphamide was restarted. Combination MMF and tacrolimus did not maintain control of disease and renal function continued to decline. Belimumab IV-monthly was added in combination and proteinuria halved, with stabilisation of renal function, although now with eGFR 25ml/min. The most recent biopsy shows chronic damage. SLEDAI-improved from 22 to 10.

**Case report - Discussion:**

In children, adolescent females are predominantly affected as observed in this case. The peak age of onset is around 12 years, often with more severe disease presentation than lupus in adults; with a higher incidence of major organ involvement and aggressive disease course with a higher chance of developing complications.

She developed uncontrolled hypertension and nephrotic syndrome requiring treatment with multiple antihypertensive agents and thrombosis prevention with DOACs. The patient was offered but refused anaemia treatment with erythropoietin. She received infection prevention treatment (PCP prophylaxis) and GnRH agonist injections to preserve ovarian function while on cyclophosphamide.

Later, she developed iatrogenic cushingoid syndrome and severe facial acne. Poor compliance, commonly seen in this age group, possibly contributed to the poor outcomes.  Other medication-related complications included retinal hydroxychloroquine toxicity that led to dose reduction. Allergy to rituximab (Mabthera) prevented its further use; given the severity of reaction concerns, other biosimilar humanized anti-CD20 antibodies were considered, but difficult to obtain; deteriorating renal function with predominantly damaged kidneys and class V nephritis on biopsy and aiming to control immunology along with other active symptoms (arthritis, serositis–pleurisy, systemic symptoms, anaemia) options were limited. There was increasing evidence to use belimumab plus tacrolimus +/- Mycophenolate-Mofetil to maintain remission. Belimumab was used after a repeat course of cyclophosphamide for this patient, after discussion with tertiary renal centres.

During the course of her illness, she had progressively deteriorating renal function, which prompted discussion for renal replacement therapy or renal transplant. Considering that this was affecting her at such a young age, she was advised to keep an up-to-date vaccination status. Her worsening condition as well as its associated complications related to treatment and polypharmacy have significantly affected her mental health and quality of life in many ways, including suffering from depression and fatigue. Frequent hospital visits for investigations and treatment also hampered her education. This emphasizes the importance of social, family, and educational support for patients with this condition.

In conclusion, despite a multidisciplinary approach and access to specialist services severe relapsing course of SLE and multiple diseases and treatment-related complications developed with co-morbidity, leading to life-changing organ damage and impact on quality of life.

**Case report - Key learning points:**

>

1. In paediatric presentations, vague symptom onset and presentation to general practice and general paediatrics with a rare condition can lead to delay in initial diagnosis. She presented initially with multi-organ involvement, serositis, and joints involvement later despite having combination immunosuppressive therapies; having recurrent flares leading to persistent proteinuria and end-stage renal failure indicates an aggressive disease course in children.
2. Managing complications related to SLE such as anaemia, hypertension, nephrotic syndrome and depression is challenging and requires multi-disciplinary input. Clinicians need to be aware of side effects/complications of immunosuppressive therapies in children and sometimes withdrawal of offending agents can help in resolution.
3. DVT prophylaxis in high-risk lupus profile and nephrotic syndrome-lupus nephritis with anticoagulation therapy is mandatory and contraception with progesterone-only-products is recommended owing to less risk of DVT.
4. Response and bioavailability of potent immune-modulating drugs differ between adults and children. Side effects of drugs can be particularly problematic in adolescents. They can have a dramatic impact on education, social development, and the whole family.
5. If allergy precludes the use of rituximab, availability of other anti-CD20-monoclonal antibody drugs is still very limited
6. There is increasing evidence that belimumab (monoclonal antibody) can also have a benefit on renal lupus.
7. Patients with lupus nephritis are at risk of chronic kidney disease and it would be appropriate to have up-to-date immunization-status in case the patient needs renal transplant consideration in the future.
8. Patients should be able to continue study and in view of symptoms and the disruption due to illness, the hospital should offer support letters to schools/universities to enable flexibility with assignments and time extension.

To conclude, jSLE is a challenging disease that is both difficult to diagnose and to treat. Clinicians should be aware of the greater risk of systemic complications in children with SLE.

